# Assigned NMR backbone resonances of the ligand-binding region domain of the pneumococcal serine-rich repeat protein (PsrP-BR) reveal a rigid monomer in solution

**DOI:** 10.1007/s12104-020-09944-9

**Published:** 2020-04-20

**Authors:** Tim Schulte, Benedetta Maria Sala, Johan Nilvebrant, Per-Åke Nygren, Adnane Achour, Andrey Shernyukov, Tatiana Agback, Peter Agback

**Affiliations:** 1 Science for Life Laboratory, Department of Medicine, Solna, Karolinska Institute, and Division of Infectious Diseases, Karolinska University Hospital, SE-171 76 Stockholm, Sweden; 2 Division of Protein Engineering, Department of Protein Science, School of Engineering Sciences in Chemistry, Biotechnology and Health, AlbaNova University Center, Royal Institute of Technology, and Science for Life Laboratory, SE-100 44 Stockholm, Sweden; 3 Department of Molecular Sciences, Swedish University of Agricultural Sciences, PO Box 7015, 750 07 Uppsala, Sweden; 4 Laboratory of Magnetic Radiospectroscopy, N.N. Vorozhtsov Institute of Organic Chemistry, SB RAS, Lavrentiev ave. 9, Novosibirsk, Russia 630090

**Keywords:** NMR assignments, Pneumococcal serine rich repeat protein, Secondary structure, X-ray comparison, Backbone dynamics

## Abstract

The pneumococcal serine rich repeat protein (PsrP) is displayed on the surface of *Streptococcus pneumoniae* with a suggested role in colonization in the human upper respiratory tract. Full-length PsrP is a 4000 residue-long multi-domain protein comprising a positively charged functional binding region (BR) domain for interaction with keratin and extracellular DNA during pneumococcal adhesion and biofilm formation, respectively. The previously determined crystal structure of the BR domain revealed a flat compressed barrel comprising two sides with an extended β-sheet on one side, and another β-sheet that is distorted by loops and β-turns on the other side. Crystallographic B-factors indicated a relatively high mobility of loop regions that were hypothesized to be important for binding. Furthermore, the crystal structure revealed an inter-molecular β-sheet formed between edge strands of two symmetry-related molecules, which could promote bacterial aggregation during biofilm formation. Here we report the near complete ^15^N/^13^C/^1^H backbone resonance assignment of the BR domain of PsrP, revealing a secondary structure profile that is almost identical to the X-ray structure. Dynamic ^15^N-T_1_, T_2_ and NOE data suggest a monomeric and rigid structure of BR with disordered residues only at the N- and C-termini. The presented peak assignment will allow us to identify BR residues that are crucial for ligand binding.

**Electronic supplementary material:**

The online version of this article (10.1007/s12104-020-09944-9) contains supplementary material, which is available to authorized users.

## Biological context

The Gram-positive commensal and human-adapted bacterium *Streptococcus pneumoniae* colonizes the upper respiratory tract in about 10% of healthy adults and up to 60% of children, without necessarily causing any symptoms (van der Poll and Opal 2009). However, upon certain not yet well-defined triggers, pneumococcus is transformed from a silent colonizer to a virulent pathogen causing diseases such as otitis media, sinusitis, pneumonia, septicemia and meningitis (Weiser et al. 2018). For efficient colonization within the nasopharynx, pneumococcus displays a multitude of surface-exposed modular adhesive and catalytic proteins (Perez-Dorado et al. 2012).

One such adhesin is the giant pneumococcal serine rich repeat protein (PsrP), that was discovered as a key colonization factor present in 60% of pneumococcal strains capable of causing pneumonia in children (Blanchette-Cain et al. 2013; Sanchez et al. 2010). PsrP belongs to a family of serine rich repeat proteins (SRRP) displayed on the surface of Gram-positive bacteria for bacterial attachment to host cells (Lizcano et al. 2012). The C-terminal LPXTG motif of PsrP is recognized by Sortases and covalently linked to the bacterial peptidoglycan cell wall. SRRPs share a long, highly repetitive and glycosylated C-terminal serine rich-repeat (SRR) region that varies in length between 400 and 4000 residues. Their functional binding region (BR) domains, which bind to a broad range of targets including extracellular DNA (eDNA), glyco-conjugates and keratins, are variable in sequence and organized into modular domains (Lizcano et al. 2012).

**Fig. 1:**
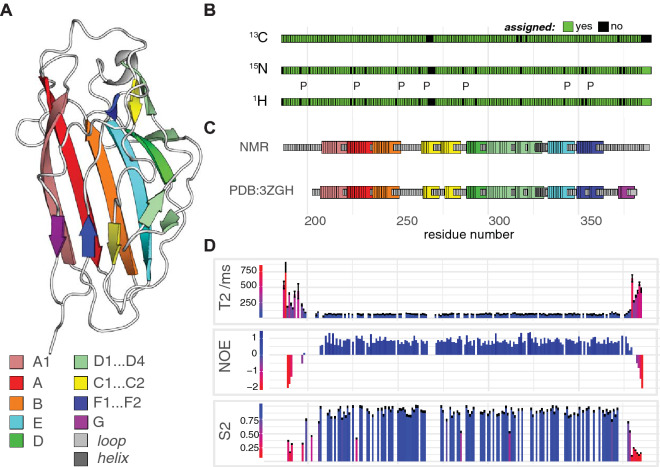
Near complete ^15^N/^13^C/^1^H backbone resonance assignment of BR_187–385_ revealed a rigid monomer with secondary structure almost identical to the X-ray structure (PDB:3ZGH). **a** The crystal structure of BR_187–385_ is visualized as in Fig. 3 of our previous publication (Schulte et al. 2014). **b** Assigned non-proline backbone ^15^N and ^1^HN as well as ^13^Cα are shown in green. Non-assigned residues are shown in black. Proline residues are highlighted (P). **c** The secondary structure of BR_187–385_ calculated from the NMR chemical shifts was compared to the crystal structure at the residue-level, and color-coded according to the cartoon representation in panel **a**. Prior to the secondary structure calculations, random coil chemical shifts of BR_187–385_, were calculated using POTENCI (results shown in Figure S1) (Nielsen and Mulder 2018; Schwarzinger et al. 2000). **d** T_2_ relaxation times, ^15^N-NOEs data as well as the general order parameter S^2^ reveal a rigid barrel with flexible N- and C-termini. Panels **b**–**d** are visualized on a common axis corresponding to the residue numbers of BR_187–385_. T_1_ relaxation times are shown in Figure S2

The positively charged BR domain of PsrP binds to negatively charged helical structures such as keratin-10 (KRT-10) and eDNA, possibly to promote efficient bacterial attachment to the upper respiratory tract and during biofilm formation, respectively (Sanchez et al. 2010; Shivshankar et al. 2009; Blanchette-Cain et al. 2013; Schulte et al. 2014, 2016). The crystal structure of the KRT10- and DNA-binding domain of PsrP (BR_187–385_) revealed a fold topology that is distantly related to the adhesin CnaA of *S. aureus*, a microbial surface component recognizing adhesive molecule (MSCRAMM) (Deivanayagam et al. 2002; Schulte et al. 2014) (Fig. 1a). MSCRAMMs were defined by a common ligand binding mechanism that is mediated by two adjacent subdomains comprising Ig-like folds (Foster et al. 2014). In the structurally and mechanistically well-described ‘dock, lock and latch’ (DLL) binding mode, extracellular matrix-derived peptide ligands dock to the open apo form of MSCRAMMs and conformational changes create a closed form, in which the ligands are locked into place (Foster et al. 2014). Most of the described DLL and associated binding mechanisms were derived from X-ray structures of apo- and ligand-bound forms of MSCRAMMs, and in-depth biophysical investigations revealed strong interactions even withstanding forces in the covalent bond range (Deivanayagam et al. 2002; Xiang et al. 2012; Ross et al. 2012; Milles et al. 2018; Herman et al. 2014).

While we have presented structural docking models for binding of KRT-10 or eDNA to the BR domain of PsrP, neither their complex structures nor defined molecular binding mechanisms have been demonstrated. It is also yet unclear whether the previously identified inter-molecular β-sheet dimer of BR is required for ligand binding. In this report we present the complete ^1^H, ^13^C and ^15^N backbone assignment of the PsrP-BR domain that will allow us to identify BR residues that are crucial for ligand binding and involved in binding-associated conformational dynamics. Our on-going ligand titration experiments will reveal if BR adopts a binding mode similar to or different from the DLL mode of MSCRAMMs.

## Methods and experiments

### Protein production

A TEV-cleavage site (ENLYFQG) was inserted between the poly-His tag and the BR domain of the previously described BR^187−385^ construct using sequence and ligation-independent cloning to yield His_6_-TEV-BR^187−385^ (Li and Elledge 2007; Schulte et al. 2014). The protein was expressed heterologously in *E. coli* and purified as previously described (Schulte et al. 2014). Single ^15^N and double ^15^N/^13^C labeled proteins were expressed in minimal M9 medium according to published protocols (^15^NH_4_Cl: 1 g/L and ^13^C d-glucose: 10 g/L) (Sivashanmugam et al. 2009; Fox and Blommel 2009). Bacteria were grown to an OD of about 0.4–0.7 in 4L TB medium, pelleted and re-suspended in 400 mL of minimal NMR-growth medium for o/n expression at 25 °C.

All purification buffers were based on 20 mM HEPES, 300 mM NaCl, 10% glycerol pH 7.5. His_6_-TEV-BR^187−385^ was purified using immobilized metal affinity (IMAC, HisTrap FF GE-Healthcare) and size exclusion chromatography (SEC, Superdex 75, GE Healthcare). The poly-His tag was removed by TEV cleavage in HEPES buffer comprising 5 mM EDTA and 1 mM DTT. Cleaved protein was passed through the Ni-NTA column as flow-through. The final sample was purified over Superdex 75 column in 50 mM sodium-phosphate buffer pH 5.0, 100 mM NaCl, and concentrated using centrifugation ultrafiltration.

### NMR samples preparation

All NMR experiments were performed in the buffer containing 50 mM Na–phosphate pH 5.0, 100 mM NaCl, with added 1 mM NaN_3_ and 10 (v/v) % D_2_O. The buffer-exchanged protein was concentrated to at least 0.7 mM using centrifugation ultrafiltration and 280 μL was placed in a 5 mm shigemi tube.

### NMR spectroscopy

NMR experiments were acquired on Bruker Avance III spectrometers operating at 14.1 T, equipped with a cryo-enhanced QCI-P probe at a temperature of 298 K. The backbone residues were assigned, based on standard 3D TROSY triple resonance experiments. The iterative non-uniform sampling protocol (NUS) comprised HNCO, HNCA and HN(CO)CA, HN(CA)CO, HN(CO)CACB and HNCACB experiments (Jaravine et al. 2008; Orekhov and Jaravine 2011; Salzmann et al. 1998, 1999). A 25% sampling schedule was used for all other 3D spectra, yielding a total acquisition time of 153 h (about 1 week). Targeted acquisition (TA) was used for automatic processing and analysis of spectra as described previously (Jaravine and Orekhov 2006; Jaravine et al. 2008; Orekhov and Jaravine 2011). This novel procedure reduces significantly data acquisition and analysis time to assign backbone resonance peaks of proteins (Unnerstale et al. 2016; Agback et al. 2019). The automatic assignment was validated manually using CcpNmr Analysis 2.4.2 (Vranken et al. 2005).

H^α^ protons were assigned using a 3D HCACO sampling schedule comprising 25% NUS and ^15^N-hsqc-NOESY, ^13^C-hsqc-NOESY (Kay et al. 1990, 1992; Schleucher et al. 1994). Data were processed and assigned by Topspin 4.0.6 (Bruker) and CcpNmr Analysis 2.4.2, respectively (Vranken et al. 2005). The ^1^H, ^13^C and ^15^N backbone chemical shifts were referred to DSS-d6 directly, while ^13^C and ^15^N chemical shifts were referred to indirectly.

Random coil chemical shifts of BR_187–385_, were calculated using POTENCI with neighbour correction and subtracted from the experimental ^1^HN, ^15^N, ^13^Cα, ^13^Cβ, ^13^C′ and H^α^ chemical shifts (Nielsen and Mulder 2018; Schwarzinger et al. 2000) (Figure S1). The chemical shift index (CSI) was calculated according to the original method (Wishart et al. 1992). Residues with consecutive Δδ^13^C′ or Δδ^13^Cα values above 0.7 ppm and below − 0.7 ppm indicate alpha helix, and beta strands, respectively. The opposite is valid for Δδ^13^Cβ. The CSI for the three nuclei were averaged and reported as “consensus” CSI.

T_1_, T_2_ and NOEs were determined using sensitivity enhanced TROSY-type pulse-sequences with temperature compensation train of pulses after acquisition time (Zhu et al. 2000). T_1_ relaxation was determined from the following series of relaxation delays: 10, 90, 192, 320, 480, 690, 980, 1220 and 1444 ms. T_2_ relaxation was measured using *CPMG* delays of 8.5, 17.0, 25.4, 33.9, 42.4, 50.9, 59.4, 76.3 and 93.3 ms. Both T_1_ and T_2_ experiments were repeated to estimate the experimental fitting to about 2%. The same error was set for the NOE experiment. All spectra were processed by Topspin 4.0.6 (Bruker) and evaluated using Dynamics Center 2.1 (Bruker), in which T_1_ and T_2_ data were fit using mono exponential decay functions. NOEs were obtained by dividing the ^1^H–^15^N peak intensities in a NOE-enhanced spectrum by the corresponding intensities in an unsaturated spectrum. The order parameters, S^2^, and the fast internal correlation time, τ_E_ were obtained by fitting the relaxation parameters at one field using the Lipari–Szabo model-free approach with a NH bond length of 1.02 Å and a CSA of − 160 ppm (Hiyama et al. 1988; Lipari and Szabo 1982a, b).

In the figures and in the text, the standard nomenclature for amino acids of the carbon atoms was used, where ^13^Cα is the carbon next to the carbonyl group ^13^C′ and ^13^Cβ is the carbon next to ^13^Cα (Markley et al. 1998). The secondary structures obtained from NMR and X-ray crystallography were compared using CSI 3.0 (Hafsa et al. 2015). The crystal structure and NMR data presented in Fig. 1 were visualized using PyMol and the R tidyverse, respectively (Schrödinger 2010; Wickham 2016, 2017).

### Extent of assignments and data deposition

**Fig. 2:**
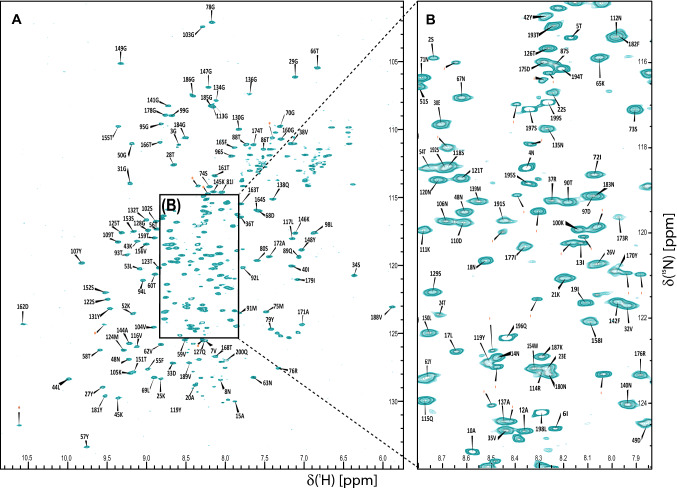
^1^H–^15^N TROSY spectrum of BR_187–385_ with assigned residue numbers. Cross peaks labelled with red * belong to multiple conformations of N- and C-terminal residues

Targeted Acquisition (TA) and conventional approaches were combined to assign 94% of non-proline backbone ^15^N and ^1^HN, 98% of ^13^Cα, 96% of ^13^Cβ, 96% of ^13^C′ and 91% of non-glycine H^α^ (Fig. 1b). All assigned chemical shifts are labelled in Fig. 2 and the associated peak table has been deposited to BMRB with accession code 50157. The secondary structure profile derived from the NMR data was almost identical to the previously determined crystal structure, thus validating our resonance assignment (Fig. 1a, c). In more detail, the previously determined crystal structure of BR_187–385_ revealed a fold that is distantly related to the DEv-IgG fold topology of MSCRAMMs (Schulte et al. 2014). The DEv-IgG fold topology can be described as a compressed barrel composed of two opposing β-sheets that are formed by β-strands ABED (sheet I) and CFG (sheet II), and is distinguished from the IgG-fold by the insertion of two extra strands between strands D and E (Deivanayagam et al. 2002; Vengadesan and Narayana 2011). In BR_187–385_, the CFG sheet is heavily distorted by loops and β-turns, and four strands (D1 to D4) are inserted between strands D and E (Fig. 1a). The NMR assignment revealed that residues T353-A356 between strands F1 and F2 adopted a β-strand in solution, thus combining the two short strands into a single F strand comprising nine residues (Fig. 1a, c). Subtle differences between the crystal and NMR secondary structures were noticed for turn and disordered regions following strand F. The two short strands C1 and C2 were each extended by two residues in solution, but a short sequence comprising residues Y267-G270 in between the two strands was not assigned. Furthermore, the short G-strand was identified as unstructured in solution. The N- and C-termini comprising G186-E208 and T359-Q385 respectively, were identified as unstructured.

Dynamic backbone motions of BR_187–385_ on a pico- to nanosecond timescale were evaluated by determining the longitudinal (^15^N T_1_) and transverse (^15^N T_2_) relaxation times as well as steady-state heteronuclear nuclear Overhauser enhancement (^15^N NOE) of each ^1^H–^15^N amide bond (Figs. 1 and S2). High T_2_ relaxation times, large negative ^15^N-NOE values and low general order parameter S^2^ values indicated highly dynamic N- and C-termini of BR_187–385_ (Fig. 1d). Indeed, these regions were not observed in the previously determined crystal structure. However, low T_2_ relaxation times, positive ^15^N-NOE values as well as S^2^-values between 0.8 and 1 revealed a rigid structure of the compressed BR_187–385_ barrel (Fig. 1). Furthermore, the correlation time for molecular reorientation (τ_c_) was estimated to 13 ns as expected for a 20 kDa protein, indicating that in solution BR_187–385_ is a monomer.

In conclusion, the near complete ^15^N/^13^C/^1^H backbone resonance assignment of BR_187–385_ revealed a secondary structure profile almost identical to the X-ray structure. BR_187–385_ was monomeric and rigid in solution exhibiting disordered flexible N- and C-termini. Studies of the structure and dynamics of the BR_187–385_ in complex with ligands in solution are on-going and will provide important insights in the molecular bases underlying these interactions.

## Electronic supplementary material

Below is the link to the electronic supplementary material.

Supplementary file1 (PDF 295 kb)
