# Supplementary material for: Assigned NMR backbone resonances of the ligand-binding region domain of the pneumococcal serine-rich repeat protein (PsrP-BR) reveal a rigid monomer in solution
Source: Biomol NMR Assign. 2020 Apr 20;14(2):195–200. doi: 10.1007/s12104-020-09944-9 (PMC7462905; doi:10.1007/s12104-020-09944-9)
Supplement: Supplementary file 1 — Supplementary file1 (PDF 295 kb) [file 12104_2020_9944_MOESM1_ESM.pdf]

Assigned NMR backbone resonances of the ligand-binding region domain of the pneumococcal Serine-Rich Repeat Protein (PsrP-BR) reveal a rigid monomer in solution

Tim Schulte[1], Benedetta Maria Sala[1,2], Johan Nilvebrant[2], Per-Åke Nygren[2], Adnane Achour[1], Andrey Shernyukov[3, 4], Tatiana Agback[3] and Peter Agback[3, *]

[1] Science for Life Laboratory, Department of Medicine Solna, Karolinska Institute, and Division of Infectious Diseases, Karolinska University Hospital, Solna, Stockholm, Sweden

[2] Division of Protein Engineering, Department of Protein Science, School of Engineering Sciences in Chemistry, Biotechnology and Health, AlbaNova University Center, Royal Institute of Technology, Stockholm, and Science for Life Laboratory, Solna, Sweden.

[3] Department of Molecular Sciences, Swedish University of Agricultural Sciences, PO Box 7015, SE-750 07 Uppsala, Sweden.

[4] Laboratory of Magnetic Radiospectroscopy, N.N. Vorozhtsov Institute of Organic Chemistry, SB RAS, Lavrentiev ave. 9, Novosibirsk, 630090, Russia

* Corresponding author, peter.agback@slu.se

Supplemental figures

CS for Cα

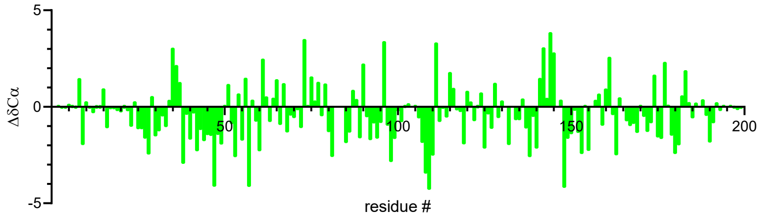

CS for Cβ

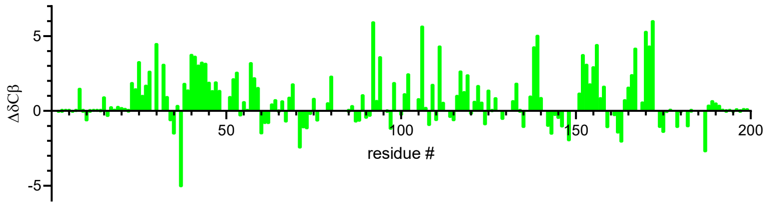

CS for CO

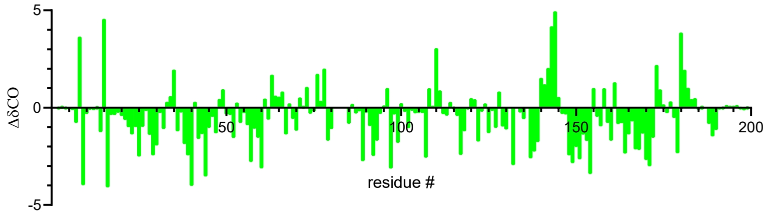

CS for N

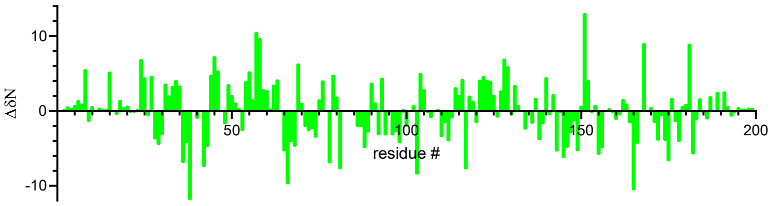

CS for HN

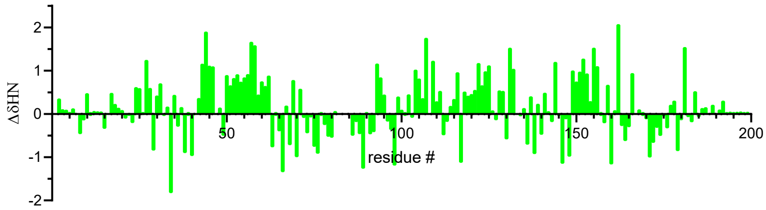

CS for Hα

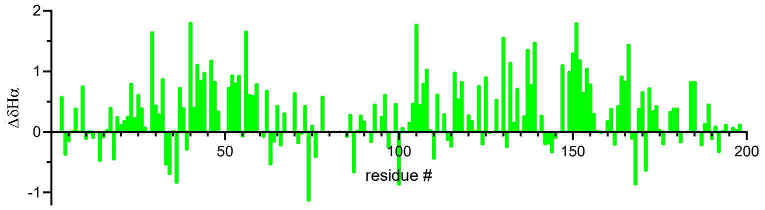

Figure S1 Differences of the chemical shifts (Δδ) of the ^{1}HN, ^{15}N , ^{13}Cα, ^{13}Cβ, ^{13}C′ and H^{α} nuclei obtained as difference between the assigned experimentally chemical shifts and the random coil chemical shifts with neighbour correction factor in POTENCI (Nielsen & Mulder, 2018) as described in the method. The discrimination thresholds of the differences to be used for determination of CSI of secondary structure are 0.1 ppm for ^{1}Hα, 0.7ppm for ^{13}Cα and ^{13}Cβ, and 0.5ppm for ^{13}C′ as accepted from (Mielke & Krishnan, 2009).

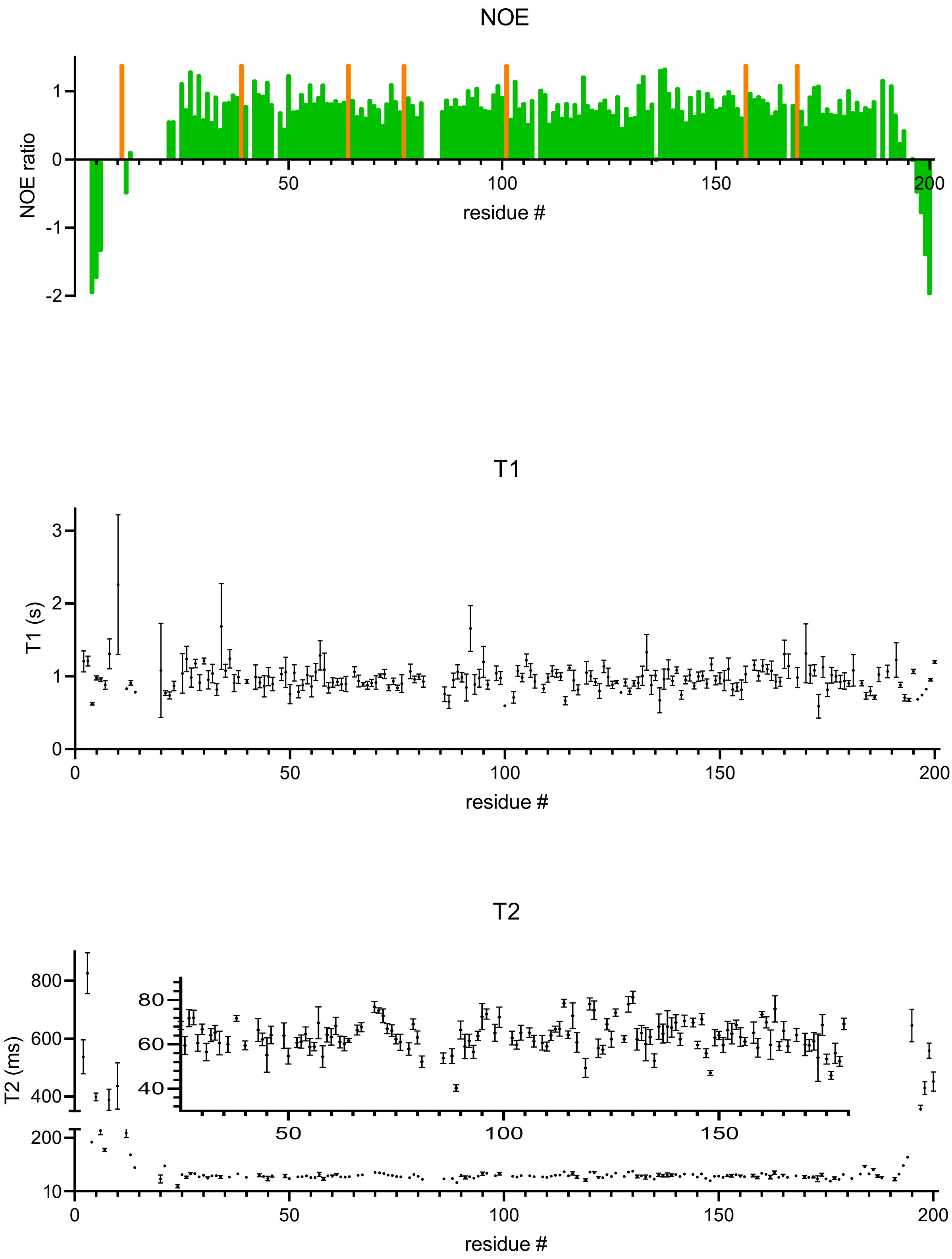

Figure S2 Longitudinal (T_1) and transverse (T_2) relaxation times and steady state heteronuclear ^{15}N-NOEs of $BR_{187–385}$. Obtained from Protein dynamics center 2.5.5 (Bruker). The orange bars are prolines.
